# Fixed-time artificial insemination technology in buffaloes: a review

**DOI:** 10.3389/fvets.2025.1586609

**Published:** 2025-06-02

**Authors:** Hubdar Ali Kolachi, Xiaomeng Zhang, Muhammad Mohsen Rahimoon, Muhammad Shahzad, Ayantoye Jesse Oluwaseun, Muhammad Ibrahim Panhwar, Mohammad Farooque Hassan, Omaima Mohammad Tawfik Kandil, Pengcheng Wan, Xueming Zhao

**Affiliations:** ^1^State Key Laboratory of Sheep Genetic Improvement and Healthy Breeding, Institute of Animal Husbandry and Veterinary Sciences, Xinjiang Academy of Agricultural and Reclamation Sciences, Shihezi, China; ^2^Institute of Animal Sciences (IAS), Chinese Academy of Agricultural Sciences (CAAS), Beijing, China; ^3^Shaheed Benazir Bhutto University of Veterinary & Animal Sciences, Sakrand, Sindh, Pakistan; ^4^Department of Animal Reproduction & AI Veterinary Research Division, NRC, Cairo, Egypt

**Keywords:** buffalo, estrous synchronization, reproductive cycle, TAI, FTAI protocols, fixed-time artificial insemination (FTAI)

## Abstract

Buffalo occupies a leading position as a major livestock commodity and is the primary milk-producing animal in many countries like Italy, China, India, Pakistan, Bangladesh, and Nepal. Buffalo farming emphasizes the significance of effective reproductive strategies. Among effective reproductive strategy, artificial insemination has a significant influence on herd's genetic progress. Nonetheless, buffaloes exhibit unique reproductive behavior, which complicates the insemination process. These animals demonstrate inconsistent periods (ranging from 6–48 h) of mounting acceptance. Therefore, timed artificial insemination (TAI) has surfaced as a useful technique for advancing buffalo breeding initiatives and omits the need for heat detection. TAI enhances reproductive management and genetic progress in buffaloes by synchronizing estrus and optimizing insemination timing. This review focuses on examining buffalo reproductive physiology, particularly emphasizing estrus synchronization protocols, ovulation, and TAI. We also provide a brief description of the factors influencing TAI success, such as hormonal treatments and environmental conditions. This review underscores TAI's importance identifies areas for further research and development and reinforces its central role in sustainable buffalo farming.

## 1 Introduction

Buffaloes are crucial dairy animals commonly found in warm regions. Over the past 37 years, their global population has notably grown, surpassing 205 million by 2025, 98% of which are found in Asia, 0.7%−0.8% in Africa remarkably in Egypt, 1% and in South America, and Europe 0.2% (1, 2). This short-day seasonal breeder animal shows increased activity as the day length decreases (3). A noticeable factor controlled by melatonin release, in addition to heat stress, is the impact of seasonal breeding patterns on buffalo reproductive activity. However, among female buffaloes, younger animals, such heifers, have a more uniform reproductive function, whereas older animals are more sensitive to such photoperiodic alternance in reproductive efficiency (4). Any disruptions in the reproductive organs that occur at the end of the good reproductive season, between the end of summer and the end of the following winter, and until spring at latitudes above the equator, will actually cause anaetrus in older animals (5). Although melatonin is released all year long, the photoperiod determines how long it lasts, which influences gonadotropin and steroidogenesis, which is a seasonal breeding phenomena in buffaloes (6, 7). By activating its receptors (MTNR1A and MTNR1B) and binding sites in the HPG hypothalamic–pituitarygonadal (HPG) axis, melatonin contributes to sexual development and the restoration of ovarian functions. Melatonin enhances calcium ion influx to GnRH-expressing neurons in GnRH secretion and controls the HPG axis by varying the expression of the gonadotropin gene according to the season (8). This indicates the origin of Buffalo domestication in the Indus Valley (Moen-Jo-Daro) Civilization where calving was synchronized with favorable climatic conditions and abundant food resources marking the early stages of buffalo domestication (9–11). Additionally, while being polyestrous, buffaloes' reproductive effectiveness varies greatly during the year. Buffaloes that give birth during an unfavorable season might not continue ovarian activity until the next advantageous season since buffalo cows show a clear seasonal variation in showing estrus, conception rate, and calving rate (12). When AI is applied outside of the breeding season, the pregnancy rate is impacted by this seasonal reproductive rhythm. The application of AI in buffaloes may increase if hormonal therapies can mitigate some of the challenges associated with seasonality and estrus detection (13). These animals have a unique role in rural livestock farming, particularly in Asia, where their productivity greatly affects the local economy (2, 14), necessitating improvements in reproductive strategies to augment food security, boost farmer income, and foster sustainable rural development (15). Buffaloes add around 13% of the world's milk production (16), with a yearly growth rate exceeding 3.5%, outpacing cow milk production, which has grown by 2.1% (17). India, Pakistan, China, Nepal, and Egypt are the countries with the largest numbers of dairy buffaloes. There are more dairy buffaloes than dairy cows in Nepal and Pakistan (1). Despite buffaloes ability to consume lower-quality food, withstand harsh environments, and resist certain diseases, enhancing the reproductive efficiency of buffalo remains a challenge (18). Silent heat is one of the challenging problems in buffaloes which affects calving intervals due to the failure of the heat detection (19). The efficiency artificial insemination programs in buffalo are highly influenced by heat detection (17).

Artificial insemination is a widely practiced reproductive technique that plays a crucial role in modern livestock breeding programs (20, 21). By enabling the controlled breeding of animals, this technology offers several benefits over natural mating, such as genetic improvement, disease control, and efficient utilization of superior sires (22, 23). In the 1930s, the introduction of AI in cattle raised questions about the optimal timing of insemination (24). Pioneering experiments focused on the ovulation window and insemination timings in dairy cattle demonstrated that the optimal artificial insemination timing is ~12–15 h after the onset of standing heat (25, 26). This groundbreaking discovery established the basis for the AM-PM rule, proposing that a cow detected in standing heat in the morning should undergo artificial insemination in the late afternoon or evening, and conversely, if detected in the evening, insemination should be performed in the morning (27, 28). The AM-PM rule allows adequate time for sperm capacitation and reaching the proper fertilization site in the oviduct (29–31). Given the anatomical and physiological similarities between cattle and water buffalo, AI methods adopted from cattle protocols were applied to water buffalo without proper consideration of ovulation timing (32, 33). The decline in the pregnancy rate due to these practices has decreased the faith of farmers in artificial insemination and modern assisted reproductive technologies (34). Paradoxically, it remains unclear whether the AM-PM rule applies to water buffalo, as this aspect has not yet been systematically investigated (35). Therefore, various protocols to facilitate fixed time artificial insemination (FTAI) have been established to synchronize follicular waves and ovulation within a predetermined timeframe (7, 36).

Extensive research and development concerning estrus and ovulation synchronization in cattle and buffaloes has been conducted, leveraging applications of FTAI (37–39). While extensively applied in cattle breeding its application in buffalo remains limited, particularly in Asia. Factors impacting its efficiency encompass heat stress, heat detection, semen quality, technician expertise, and timing of insemination (34, 40). Brazil's extensive studies on ovulation synchronization regarding FTAI in buffaloes reflect attempts to address estrus detection challenge (41). FTAI presents advantages by simplifying the management and synchronizing estrus cycles, promoting TAI for precise insemination timing, enhancing conception rates, and overall reproductive efficiency (41–43). Recent advancements allowing precise control of ovulation timing mark a substantial leap in improving buffalo reproductive efficiency (12). AI techniques adapted to each species will be necessary to maximize the reproductive physiology of buffalo and ensure their continued agricultural growth in the future (44). Thus, this review study aims to expand our understanding of the reproductive physiology of buffaloes, discuss the various synchronization protocols and explore the factors influencing FTAI success in buffaloes.

## 2 Reproductive physiology of buffaloes

Artificial Insemination (AI) has been made feasible due to advancements in our understanding of the reproductive physiology specific to buffaloes (45). Understanding the reproductive physiology of buffaloes is crucial for implementing effective breeding strategies, including FTAI (46, 47). Water buffaloes, scientifically known as *Bubalus bubalis*, have distinctive reproductive characteristics that play a significant role in their unique reproductive physiology as outlined in Table 1 (48). The fertility performance and reproductive efficiency of buffaloes in tropical conditions are distinctly influenced by the time of year (49). Buffalo reproduction is affected by several inherent challenges, as illustrated in Table 2 (50). Buffaloes show same advantages of the FTAI and ovulation synchronization already investigated in cattle (51). However, the buffaloes exhibit unique reproductive behavior, the insemination process (39). These animals do not display any homosexual behavior during heat, ensuring the necessity of teaser bull use (45, 52). Likewise, these animals demonstrate inconsistent periods (ranging from 6–48 h) of mounting acceptance (53). Since, AI technology in buffalo is applied at the end of estrus this makes its handling and utilization more challenging (41). The implementation of assisted reproductive technologies has necessitated significant developmental efforts due to the inherent peculiarities in the reproductive physiology of buffaloes (54). These techniques, which have already been established in other species, require adaptation and standardization to suit the unique reproductive characteristics of buffaloes (55, 56).

**Table 1:** Overview of buffalo reproductive characteristics.

**Aspect**	**Description**	**References**
Estrus cycle length	21–23 days	(254)
Estrous duration	10–27 h	(39)
Estrus signs	Less noticeable compared to cattle; signs include increased restlessness, mucous discharge, and mounting behavior	(81)
Ovulation	Occurs 24–48 h after the end of estrus	(254)
Gestation period	300 days	(49)
Age at first calving	46–47 months in different breeds	(63)
Puberty	10–30 months among all breeds	(48, 103)
Breeding period	Seasonal polyestrous	(255)
Reproductive longevity	15 years higher than cattle	(256)

**Table 2:** Inherent challenges affecting buffalo reproduction.

**Challenges in buffalo reproduction**	**Description**	**References**
Delayed maturity	Buffaloes exhibit a slower rate of reaching sexual maturity compared to some other livestock species	(54)
Silent estrus	Particularly prominent during summer months, buffaloes do not exhibit clear behavioral signs of estrus, making it challenging to detect their fertile period	(257)
Extended postpartum period	Buffaloes have a prolonged interval between calving and returning to estrus, impacting the calving interval and reproductive efficiency	(258)
Ovarian quiescence	Periods of reduced ovarian activity, leading to irregular estrous cycles and decreased fertility	(259)
High incidence of dystocia	Buffaloes commonly experience difficult or prolonged labor, which can negatively impact both the dam and calf	(260)
Lower conception rates	Buffaloes often exhibit lower rates of successful conception compared to some other domesticated animals	(259)

### 2.1 Estrous cycle

**Figure 1:**
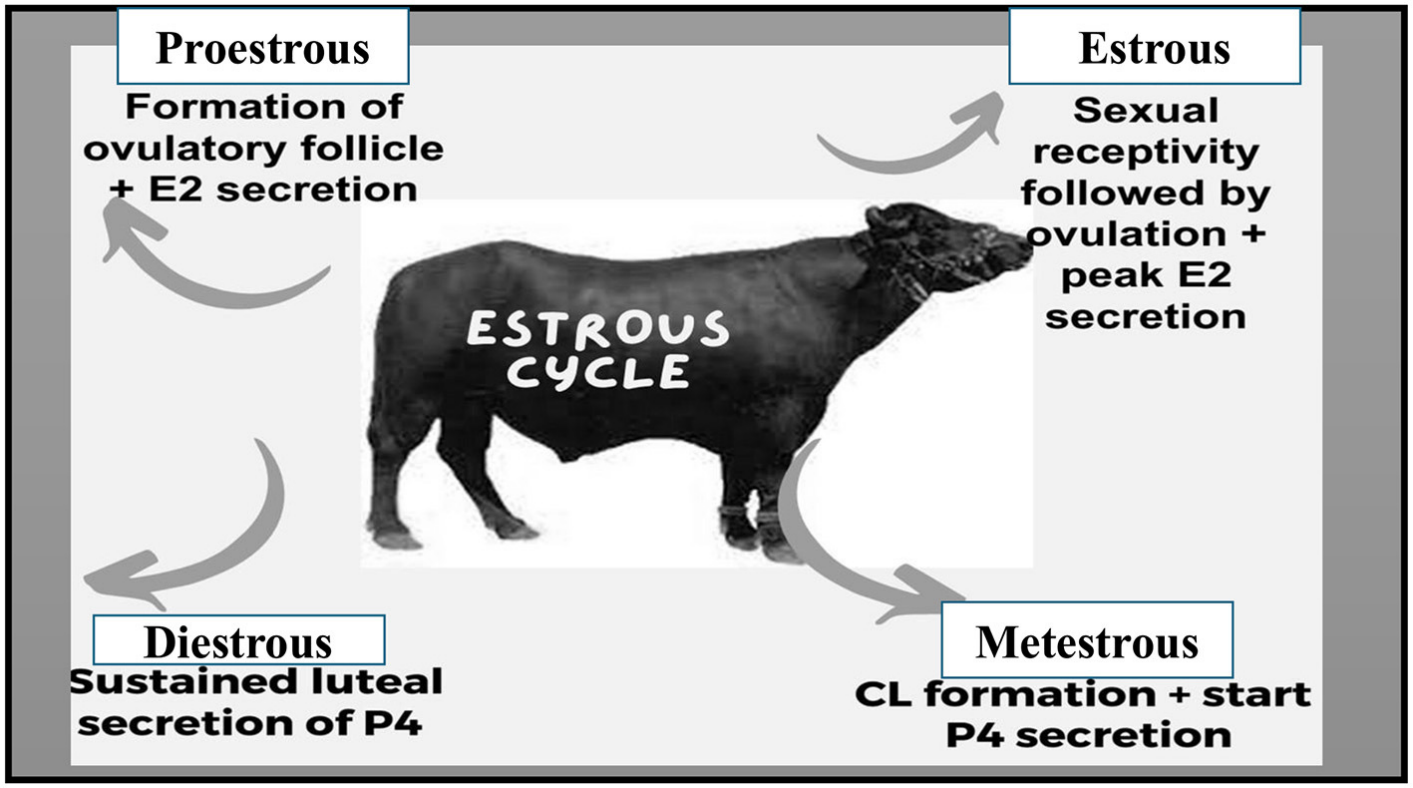
Sequential stages of the estrous cycle.

The estrous cycle encompasses the time between the end of one estrus and the onset of the next estrus (57). Water buffaloes are categorized as short-day seasonal polyestrous animals (58–60), although, under some circumstances, they can conceive all year long (61). As in the equator zone, these animals may exhibit estrous cycles throughout the year if the food supply is sufficient to sustain reproductive function (62). No significant difference has been investigated in estrous cycle length among Nilli, Murrah, local, and Crossbreeds (63). The same estrous cycle length of 21.25 ± 2.36 days was observed in Marathwada breed of buffalo (64). Estrous duration of 17–29 h. in local Nili Ravi and Murrah was seen significantly higher than the others (63). Notably, buffaloes become more seasonally polyestrous as they move farther from the equator (65, 66). The estrous cycle of buffaloes consists of two phases: the progesterone phase, also called the luteal phase, and the estrogenic phase, also called the follicular phase. There are two distinct stages in the progesterone dominant (luteal phase) i.e., metestrus and diestrus, and proestrus and estrus stages in the follicular phase as illustrated in Figure 1 (67, 68). The estrous cycle typically spans 16–33 days, with the maximum concentration occurring around days 21–24 (69), with estrus duration lasting around 12–18 h (70). Ovulation typically occurs almost 30 h after the onset of estrus, with variations ranging from 18 to 44 h (51). In contrast to cows, water buffaloes can experience heat for 8–32 h, with fewer heat symptoms (32). Buffalo follicular development follows a wave-like pattern, encompassing stages such as wave emergence (recruitment), growth, selection, dominance, and atresia in each cycle (28). The essential aspects of follicular development align with those observed in cattle (55, 71). Buffalo experience more follicular atresia compared to cattle (72). Preceding an ovulatory wave, it is common to observe 1 or 2 nonovulatory follicular waves (73, 74). Buffalo cows usually experience two to three follicular waves, with buffalo heifers commonly undergoing two-wave cycles (75, 76). Research from India (77), Brazil (78), and Pakistan (79) have indicated that greater populations of buffalo experience two follicular wave activity during the estrous cycle. The two follicular wave cycles are slightly shorter, around 21 days, compared to three-wave cycles which last about 24 days (39). In the 2nd wave dominant follicle's average size is comparable to that in the first wave i.e., 15 mm (80).

### 2.2 Estrous detection

Silent heat/estrus stands out as the primary reason contributing significantly to the diminished reproductive efficiency observed in buffaloes (81, 82). A rise in gonadotropins shortly before and during the pre-ovulatory surge of estradiol is what causes buffalo's silent heat (83). This happens because progesterone and estradiol concentrations drop during the estrus cycle. Progesterone concentrations therefore lower the peak levels of estradiol around estrus (84). Precise estrus detection is essential for efficient reproductive management, particularly during hand-mating with selected sires (85). Nocturnal behavior is one factor linked to the reduced appearance of estrus signals (86). Uncommon homosexual behavior, varying estrus duration (5–27 h), and unpredictable ovulation timing in buffaloes (24–48 h) following the heat onset are also factors affecting accurate heat detection in buffaloes (61). Traditional estrus indicators in cattle, such as swelling and redness of the vulval mucosa, vaginal mucus secretions, and repeated urination, are unreliable for water buffalo (69, 87). Behavioral manifestations of estrus, such as tail raising, bellowing, and restlessness, are observed in only a limited proportion of buffaloes and are commonly displayed during the night. Identifying precise methods for estrus detection is essential for successful re-insemination of animals that have returned to estrus (88, 89). A few techniques designed for FTAI have been developed to enhance water buffalo reproduction and eliminate the need for heat detection (90). These hormone therapies enable the regulation of luteal and follicular dynamics, estrous and ovulation (synchronization), most critically avoidance of the challenging estrus detection in this species (91–93).

### 2.3 Ovulation

The ovulation process in mammals, including buffaloes, is regulated via a complex interplay of hormones (28, 94). Compared to dairy cattle, the pre-ovulatory size of follicles in buffaloes tends to be smaller (78, 95). This is owing to lower estradiol although there may changes in the metabolism of estradiol from circulation between both the buffaloes and cattle (52). The dominant follicle's granulosa cells secrete inhibin, which suppresses the synthesis of follicle-stimulating hormone (FSH). Through inhibin release, the maturation of the dominant follicle and the consequent generation of estrogen are essential for controlling surges in luteinizing hormone (LH). An LH surge required for ovulation is caused by the dominant follicle's growing production of estradiol, which has a positive feedback effect on the pituitary and hypothalamus (96, 97). Investigations into the ovulatory responses of buffaloes after GnRH treatment in FTAI protocol have yielded valuable insights into their reproductive dynamics. It was investigated that the ovulation rate after the first GnRH shot was 60.6%. Animals ovulating after the first GnRH and ovulation treatment displayed larger dominant follicles (0.94 ± 0.17 vs. 0.67 ± 0.24 cm; *P* < 0.01) at the time of treatment compared to non-ovulating animals. Progesterone (P4) level during the initial GnRH administration did not influence ovulation rate (*P* > 0.05) (41, 98).

### 2.4 Puberty

Puberty is the stage during which reproductive organs become functionally developed, and animals gain the ability to release gametes (99, 100). For females, puberty is characterized by the age at which they experience their initial estrus, subsequently leading to ovulation (101). Buffaloes typically at 55%−60% of adult body weight (250–400 kg) attain puberty (102, 103). The age of puberty onset varies significantly, spanning from 18 to 46 months (18, 104). At favorable conditions, the riverine type attains puberty at the age of 15–18, and swamp buffalo at 21–24 months (103, 105). Nutrition, genotype, management, and climate contribute to this variation (106). Animals born in spring attain puberty at 380 kg body weight and 14 months of age (107). In a study of 2020 on 20 animals, it was found that there was a difference in the biochemical profile of delayed puberty animals than normal pubertal heifers (108). During an investigation on the effect of season on age at puberty, it was observed that combined climate factors like temperature and rainfall affected reproductive activity significantly but individual factor effect was not significant (109). Buffalo of India attains puberty at the age of 16 and 40 months but the average time for puberty is 2.5 years according to a report of the Central Institute for Research on Buffalo (110). All breeds exhibit variations in puberty from 10 to 36 months when nutrition and management factors are taken into account (48). Attaining puberty is more closely associated with body weight rather than age (101). Delayed puberty not only defers conception but also diminishes reproductive efficiency, extended calving intervals, and diminished manifestation of estrus consequently extending the unproductive phase (111). Bovine gonadotropin releasing factor significantly affected the buffalo's puberty onset, plasma progesterone concentration, and body weight (112).

### 2.5 Sexed semen's impact on buffalo reproductive dynamics

The use of sexed semen in buffaloes has significant implications for reproductive dynamics, particularly in enhancing calf sex ratios and improving genetic quality. Compared to cattle bulls, buffalo bulls have only been examined and chosen in the last few decades to furnish semen for artificial insemination. As a matter of fact, the latter have been the first to be the focus of intense selection, with the best bulls being chosen for genetic enhancement and then chosen for their semen quality and freezability. There is still a lot of variation in semen quality, even with the efforts made in recent decades to choose superior buffalo bulls in order to improve the annual genetic merit. If inferior quality semen has been found and shown to be unsuitable for freezing/thawing processing and AI, this variability may significantly impact the use of the best bulls. For instance, buffalo bulls' semen may be more vulnerable to oxidative stress because of increased lipid peroxidation, which is most likely caused by decreased antioxidant enzyme activity (6, 113). In order to ascertain whether pregnancies may be impacted in terms of early embryonic death and whether a season effect was anticipated between the transitional and high breeding seasons, the combined use of sexed semen and AI in pluriparous buffaloes was also investigated (114). Once more, it was determined in that follow-up study that the pregnancy rate is comparable for both unsorted and sorted semen. Furthermore, as compared to the opposite unsexed semen, the use of sexed semen did not change progesterone production or increase embryonic mortality.

The use of sexed semen for conception varied from 35 to 60%, depending on the dam's age and management, according to a review by Thakur and fellows (115). According to certain evaluations, the degree of conception varies between 30 and 80 percent among buffaloes with synchronized estrus and AI from many places and with varying ages (116). According to another study showed that employing sexed semen for buffaloes had an average conception rate of 42.7%, demonstrating its efficacy across breeds and parities (117). Praharani et al. after using sexed semen reported a mean conception rate of 50.7% and a calving rate of 46.2%, with variations based on agroecosystems (118). For genetic modification, sexed semen is essential because it enables the targeted production of female calves, which are frequently more profitable in the dairy industry (119). In addition to increasing buffalo productivity, the use of sexed semen safeguards native breeds against extinction due to shifting climates (120). Despite the benefits of sexed semen for genetic enhancement and reproductive management, issues including reduced blastocyst and cleavage rates in comparison to unsexed semen continue to be a worry (119). This emphasizes how further research is required to maximize its use in buffalo breeding efforts.

### 2.6 Role of hormones in FTAI

Hormones are biochemical substances produced by the endocrine glands that stimulate other organs of the body to produce chemical secretions (121, 122). These are essential regulators of buffalo reproductive functions (49). Hormonal treatments have been developed to manage luteal and follicular processes, creating opportunities for synchronizing follicle growth and ovulation, a vital aspect for timed artificial insemination during breeding and nonbreeding seasons (78, 123). Here we discuss the key hormones involved.

#### 2.6.1 Gonadotropin-releasing-hormone (GnRH)

GnRH is synthesized in the hypothalamus of the brain and induces the secretion of follicle-stimulating hormone (FSH) and luteinizing hormone (LH) from the anterior pituitary gland (124). It acts as a master regulator of reproductive hormones (125, 126). Injecting GnRH during follicular phase of the estrous cycle causes an LH surge, resulting in the ovulation of follicles larger than 9.0 mm (127) or promoting nonviable follicle luteinization, a few days later followed by the emergence of a new wave of follicle growth (128, 129). The simultaneous presence of a mature dominant follicle and corpus luteum at the time of GnRH injection in bovines has been associated with improved ovulation, synchronization, and conception rates (110, 130). Therefore, this hormone improves the conception rate as well as pregnancy rate when used in timed artificial insemination (110, 131).

#### 2.6.2 Follicle stimulating hormone (FSH)

FSH contributes to the growth and development of ovarian follicles (132, 133). This hormone acts as a green line for the follicles to recruit and selection (134, 135). FSH stimulates multiple follicles, typically only one becomes dominant (39). Most of the recruited follicles undergo atresia (136). Follicles that do not undergo atresia get selected and become dominant (137, 138). Peak FSH coincides with LH, averaging around 25 ng/ml (139). Following simultaneous pre-ovulatory surges in the gonadotropins, LH levels experience a sharp decline, while FSH level drops gradually (71). Recent studies have confirmed that in buffaloes, a transient peak of follicle-stimulating hormone in the blood initiates at every follicular wave (52). Weather can also influence peripheral FSH concentrations, with higher FSH/LH ratios during peak breeding seasons (140, 141). During the peak breeding season, the FSH/LH ratio was elevated compared to that in the intermediate and low breeding months (142). Nonetheless, the highest concentration of FSH on the day of estrus remained consistent across both hotter and cooler months (143, 144).

#### 2.6.3 Luteinizing hormone (LH)

LH plays a pivotal role in triggering ovulation in buffaloes (52). After the recruitment of follicles, this hormone is responsible for keeping follicles growing (145). LH surge leads to the release of the oocyte from the dominant follicle. Preovulatory surges in LH have been detected to be similar to those in cattle (146). Peripheral LH levels remain at basal levels throughout the reproductive cycle until the day of estrus, at which point a pre-ovulatory LH surge takes place (71). The period between LH surge and estrus onset is ~8–12 h (45, 147–149). Both of these hormones i.e., FSH and LH are under the control of GnRH which is synthesized and released by the hypothalamus (150).

#### 2.6.4 Progesterone

Following ovulation, the corpus luteum forms on the ovary and acts as an endocrine gland to secrete progesterone (12, 151). Progesterone prepares the uterus for pregnancy, creating an environment conducive to embryonic development (152). Suboptimal nutrition and high environmental temperatures can lead to extended periods of non-breeding (anestrus) in buffaloes (145). Monitoring progesterone metabolites in feces has been shown to effectively reflect corpus luteum functionality, correlating well with blood progesterone levels (153). Additionally, progesterone aids in transporting oocytes in the oviduct and supports early pregnancy, working in harmony with estrogens to stimulate mammary gland tissue growth (154).

#### 2.6.5 Estradiol 17β (E2)

Estrogens, including estradiol 17β (E2), influence the reproductive behavior of females (155). Estrogen is produced by the ovarian follicles and affects the central nervous system, leading to estrus behavior (156). Buffalo plasma E2 profiles resemble those of cattle cows with the highest concentrations before and during preovulatory gonadotropin surges followed by declining levels around the next days of the reproductive cycle (157). In buffalo, the E2 peak precedes the LH peak by a day (158, 159). Weather can influence plasma E2 concentrations, with lower concentrations in hot months compared to cooler months (142, 160). Decreased peak E2 values around estrus, along with lowered P4 concentrations, contribute to a higher occurrence of silent estrus in summer (129, 161). These hormones collaboratively regulate buffalo ovarian follicular growth, ovulation, fertilization, and pregnancy timing.

#### 2.6.6 Prostaglandin F2 alpha (PGF2-alpha)

The large and small luteal cells, two steroidogenic cell types that originate from ruptured follicular granulosa and thecal cell, respectively, make up the mature corpus luteum (162). Prostaglandin F2α (PGF2α) receptors on these large and tiny luteal cells can cause luteal regression when PGF2α binds to them. The luteal tissue undergoes structural alterations after the about 12-h functional period of luteolysis in buffalo, which is reflected in the blood's decreasing progesterone levels (163). In the presence of a responsive CL, which occurs between days 5 and 7 of the estrous cycle in heifers and days 7 to 17 in buffalo cows, prostaglandin F2 alpha and its synthetic analogs efficiently trigger luteolysis (164). Because cows with developed follicles enter estrus earlier than those with immature follicles at the time of treatment, the duration until estrus induction depends on the state of follicle development at the time of PGF2α administration. Procedures for two-dose PGF2α were created to guarantee the presence of responding CL (28). Additionally, PGF2α can shorten the voluntary waiting period, increasing total reproductive efficiency. Fallopian tube function is significantly mediated locally by prostaglandins. They participate in ovulation, fertilization, and the transportation of oocytes and embryos (165). It has been demonstrated earlier that PGF2α at 10 μg/ml markedly increased Caspase 3 expression at 72 h of culture in comparison to other dosages at 24 and 48 h (166).

## 3 Estrus synchronization

Estrous synchronization is the process of bringing a group of female animals into heat at the same time (167, 168). Estrous synchronization is crucial for coordinating the reproductive processes of multiple buffaloes within a herd (39). This can be achieved by shortening or extending the luteal (progesterone dominant) phase of the reproductive cycle (67, 169). This is accomplished by regulating the luteal phase through the use of progesterone analogs or prostaglandins (170) or managing follicular ovulation and development utilizing diverse combinations of progesterone, prostaglandins, hCG, eCG, GnRH, and estradiol (171). Numerous modern synchronization programs for buffalo have emerged, drawing from research primarily conducted in cattle (35). ES protocols in Buffalo have achieved partial success (172). However, a significant distinction between the buffalo and cattle is that buffaloes experience a pronounced reduction in breeding activity around the hot months of the year, resulting in reduced cyclic ovarian activity (101). This synchronization ensures that a significant number of females are in the desired reproductive stage simultaneously, allowing for efficient insemination procedures and increasing the chances of calving rate and conception rate (173). Instead of relying on visual estrus detection, which can be a significant challenge in buffalo cows, synchronization allows for planned and timely AI, minimizing missed opportunities for conception (174). Hormonal treatments enable the regulation of follicle dynamics and luteal cell functions, estrus, ovulation synchronization, and notably, alleviate the challenging task of estrus detection in this species (33, 35, 45, 170).

## 4 Fixed time artificial insemination

Fixed time Artificial Insemination is characterized as the process of performing AI at a programmed time following the synchronization of ovulation (175). It's a technology that facilitates AI without detection of heat (33). Understanding the ovarian follicular dynamics, as investigated by ultrasonography, advancements in understanding the hormonal profiles and endocrine control during the reproductive cycle have facilitated the commercial application of FTAI in buffalo population (41, 176). Typically, batches of animals are treated simultaneously with the FTAI according to a prearranged plan or schedule. It essentially means that you can easily determine how many animals to inseminate and when (season, month, and day) is the best time to do it (33). By following ovulation synchronization hormonal protocols, FTAI is made possible (177). This method eliminates the need for detecting estrus and enables breeders to enhance reproductive management through accurate insemination timing (178). FTAI involves several essential components and steps, including estrus synchronization, hormonal treatments, and meticulous insemination timing. Diverse protocols have been devised and applied in buffalo breeding to boost the success (35, 92). These protocols typically employ hormones like progesterone, GnRH, and prostaglandin to manipulate the estrous cycle and prompt ovulation at predetermined times (179–183). The critical interval between standing estrus and ovulation, which is crucial for artificial insemination, was found to be ~30 h in buffaloes (51, 184). The well-known AM-PM rule of insemination under field conditions, formerly developed for cattle has been widely applied to buffaloes (25). According to this, buffaloes have to be inseminated 12–15 h after detecting standing estrus (184, 185). However, there is a common misconception that the onset of heat signs is mistaken for the start of standing estrus, leading to early insemination. This premature insemination might reduce fertility because there's an 8 to 10-h gap between heat signs and actual standing estrus (186). To optimize fertility, buffalo cows should go through insemination 12 h after identifying standing estrus (usually done by observing teaser/bull behavior) otherwise 18–24 h after the heat signs (187). The presence of mucus during artificial insemination serves as an indicator for fertility and the intensity of natural estrus (188, 189). FTAI effectiveness can be enhanced by using ovsynch protocol in cyclic buffaloes during their breeding season and for off breeding season progesterone device with eCG+ GnRH/hCG can be used to enhance FTAI efficacy (41).

### 4.1 Fixed-TAI protocols

Numerous protocols based on the use of hormones that can act at different points in the hypothalamic-pituitary-ovarian axis have been developed in buffalo to control the estrous cycle and, in some cases, the timing of ovulation (45). The protocols can be classified as heat detection artificial insemination protocols (HDAI) are only PG based and P4 based protocols GnRH based protocols if combined with those protocols are categorized as FTAI protocols.

#### 4.1.1 Prostaglandin-based protocols

**Figure 2:**
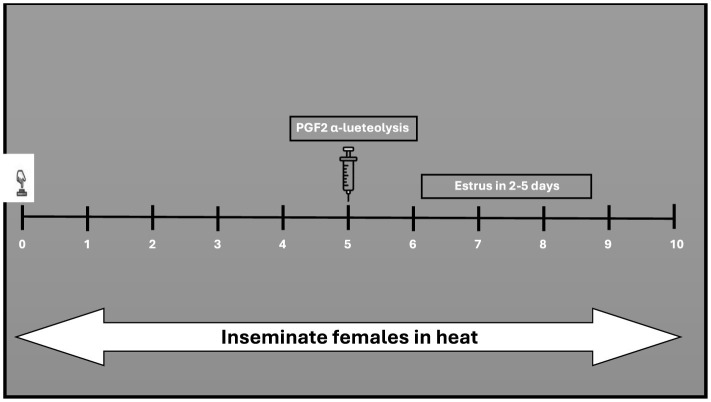
Single-shot treatment with PGF2α in the presence of an intact corpus luteum.

**Figure 3:**
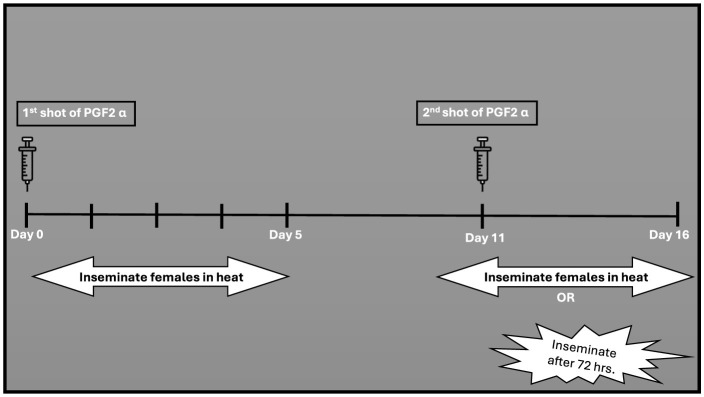
Double shot treatment of PGF2α.

This protocol shortens the luteal phase (190, 191). Prostaglandin (PGF2α) is administered to induce luteolysis (regression of the corpus luteum) in non-pregnant buffaloes (192, 193). In buffalo cows, the PGF2alpha effect is comparable to that studied in cattle (192, 194). Much like the approaches applied in cattle, buffaloes have been subjected to prostaglandin administration, either through a single injection (referred to the one-shot method also known as single shot) or through two injections apart by 11–14 days (195, 196). According to the single-shot method (Figure 2), only animals with a functional CL (5–17 days) of the estrous cycle can receive prostaglandin treatment (67). Research in cattle demonstrated that a single shot PGF2α, administered during an active corpus luteum led to estrus return in 2–3 days (39). In buffaloes, a single injection of PGF2α yielded a response alike to that observed in cattle (164). In the two-shot method, two doses of PGF2α are administered 11 days apart (Figure 3) allowing for estrus synchronization in buffaloes regardless of their specific ovarian status (195). The first injection initiates luteolysis, and the second injection ensures complete regression of the corpus luteum (197). Following the second PGF2α injection, AI is usually performed 48 to 72 h later, as ovulation is expected to occur during this timeframe (82).

#### 4.1.2 Progesterone-based protocols

**Figure 4:**
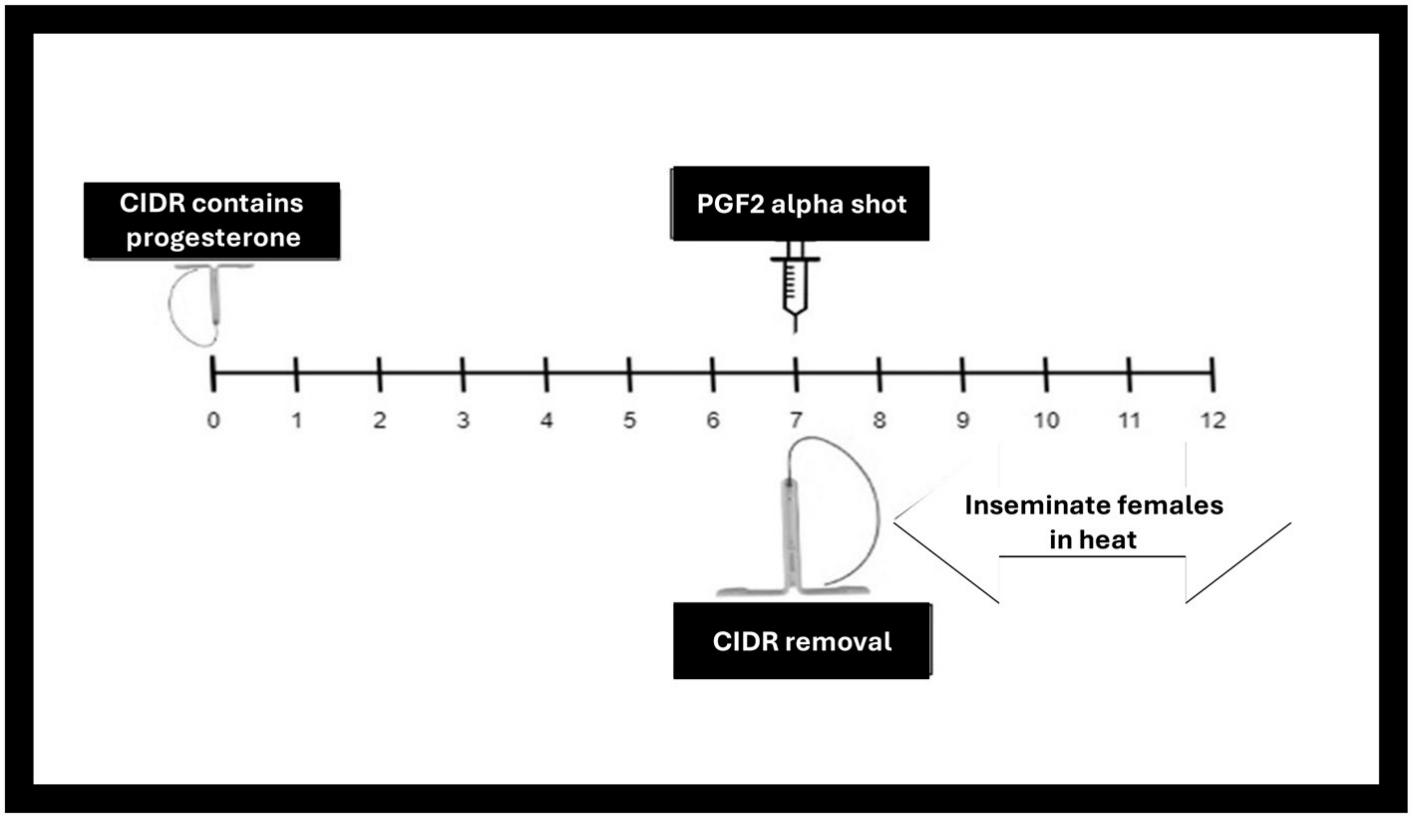
Controlled internal drug release (CIDR) progesterone-based synchronization.

This protocol lengthens the luteal phase (198). Progesterone-based treatment is used to synchronize and control the estrous cycle in buffaloes (Figure 4). One common protocol involves inserting an intravaginal progesterone-releasing device (CIDR) into the reproductive tract for a specific duration (e.g., 10–12 days) (199). It acts as an artificial corpus luteum and mimics the progesterone secretion. When it is removed after 10–12 days, the animal comes into heat (199). If at the time when CIDR is removed, an injection of PGF2α is also administered the animal comes into heat more quickly due to induced luteolysis, followed by AI within a specific timeframe, considering the expected timing of ovulation (143).

#### 4.1.3 Select synch and co synch GnRH-based protocol

**Figure 5:**
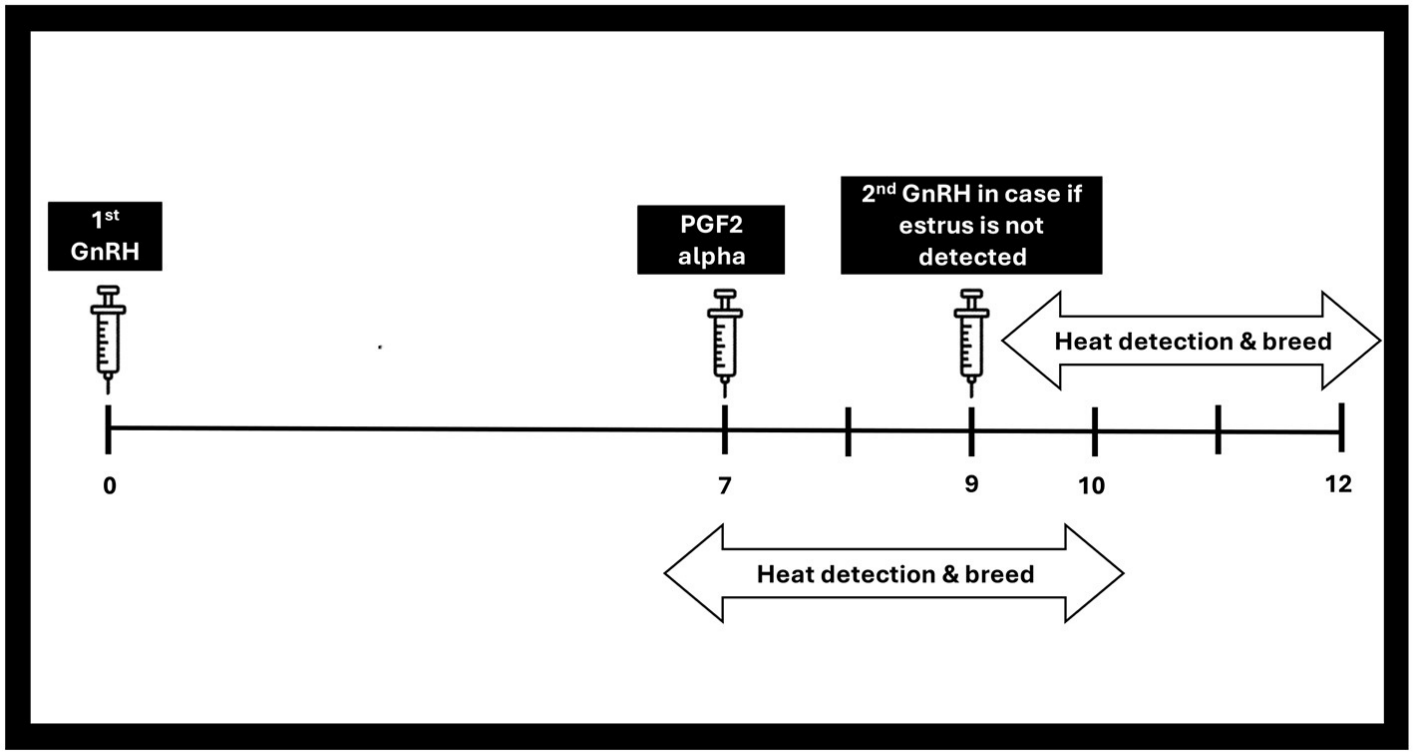
GnRH based protocol (select synch).

The initial shot of gonadotrophin-releasing hormone (GnRH) prompts ovulation in cyclic females (110), while the following injection of PGF2a facilitates the regression of the corpus luteum, leading to decreased progesterone levels (200). The second shot of GnRH injection facilitates the ovulation process from dominant follicle, which was primed by the initial GnRH treatment (67). This is illustrated in Figure 5 as well. The Co synch protocol is similar to this Ovsynch protocol except that the AI is performed at the 2^nd^ GnRH injection (146, 201).

#### 4.1.4 Ovsynch protocols

**Figure 6:**
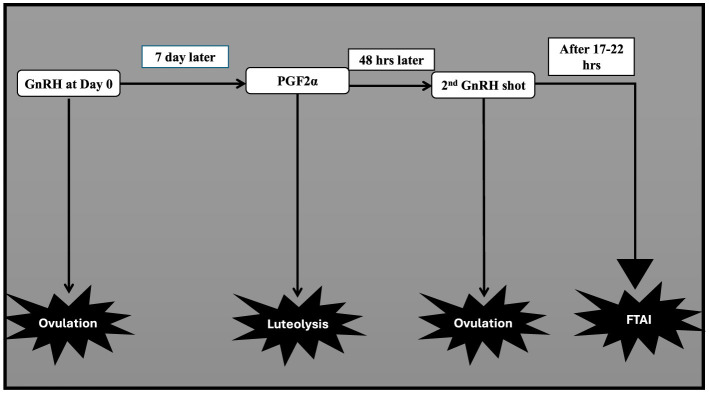
GnRH based treatment (ovsynch).

This protocol aims to synchronize ovulation in buffaloes employing GnRH and PGF2α combination (Figure 6). For the FTAI in water buffaloes, this approach has been adopted and is now the most widely used protocol, with multiple published studies supporting its use (33, 182, 202). Firstly, GnRH is administered to induce ovulation of selected dominant follicles. Then PGF2α is injected 7 days later to luteolysis and synchronize follicular wave emergence. A subsequent GnRH injection 48 h after PGF2α administration synchronized ovulation of the newly emerged dominant follicle. AI is performed at a specific time following the second GnRH injection when ovulation is expected (89, 203, 204). Table 3 presents the efficiency of this protocol in the implementation of AI without heat detection in different breeds of buffalo either riverine or swamp in various countries (205–207). The effectiveness of the treatment in buffaloes is predominantly influenced by the breeding period (38). Baruselli (65), employing the Ovsynch protocol, demonstrated a conception rate of 48.8% in buffalo cows around the breeding season and 6.9% in those inseminated during the non-breeding period. More reports utilizing the Ovsynch breeding protocol reported varying conception rates at artificial insemination varying from 56.5% during the breeding season (208) to 36.0%−42.5% during the seasonal anestrus to transition period (209, 210). The variation may be due to a greater proportion of non-cyclic animals stemming from the suboptimal activity of the hypothalamic-pituitary-gonadal axis occurring in buffalo during spring-summer (211). Indeed, studies indicate a better conception rate in cyclic compared to non-cyclic animals (212–214). According to the Ovsynch-FTAI protocol, 78%−90% of buffaloes exhibit synchronized ovulation, and 33%−60% of them get pregnant (65, 205, 209). Because of the substantial embryonic mortality (20%−40%) (215, 216) and anestrus (217) during the non-breeding season, using Ovsynch-FTAI in buffalo during these transitional periods results in a much lower pregnancy rate. When comparing pluriparous buffalo to primiparous buffalo, a greater pregnancy rate is often attained (65). Because of the low ovulatory response to first GnRH and unsynchronized development of new follicular wave Ovsynch-FTAI is not recommended in heifers (45).

**Table 3:** Ovsynch protocol for applying FTAI in buffaloes across different countries.

**Country**	**Season/ reproductive status**	**Conception rate (%)**	**References**
Bulgaria	Low breeding season	38.8	(261)
Brazil	Breeding period	48.7	(65)
	Non-breeding period	6.9	
Brazil	Breeding period/season	56.5	(208)
Romania	Breeding period/season	35–56	(183)
Nepal	Breeding period/season	64	(262)
Venezuela	Breeding period/season	35.0	(263)
Italy	Low breeding season	36.0–57.0	(209)
Nepal	Breeding period/season	46	(264)
Italy	Low breeding season	43.3	(210)
Italy	Cyclic	35.7	(212)
	Non-cyclic	4.7	
Bangladesh	Cyclic	28.0–44.4	(204)
Bulgaria	Breeding season	35.0	(265)
Egypt	Cyclic	18.0	(214)
	Non-cyclic	0.0	
Egypt	Cyclic	60.0	(213)
	Non-cyclic	35.7	
India	Cyclic	33.3	(205)
India	Low breeding season	58.1	(266)
India	Acyclic	23.1	(202)
Pakistan	Breeding season	36.3	(206)
Pakistan	Breeding season	47.0	(267)
Thailand	Breeding season	51.4	(207)
Egypt	Cyclic	59.6- 62.5	(268)

#### 4.1.5 Double-Ovsynch protocols

**Figure 7:**
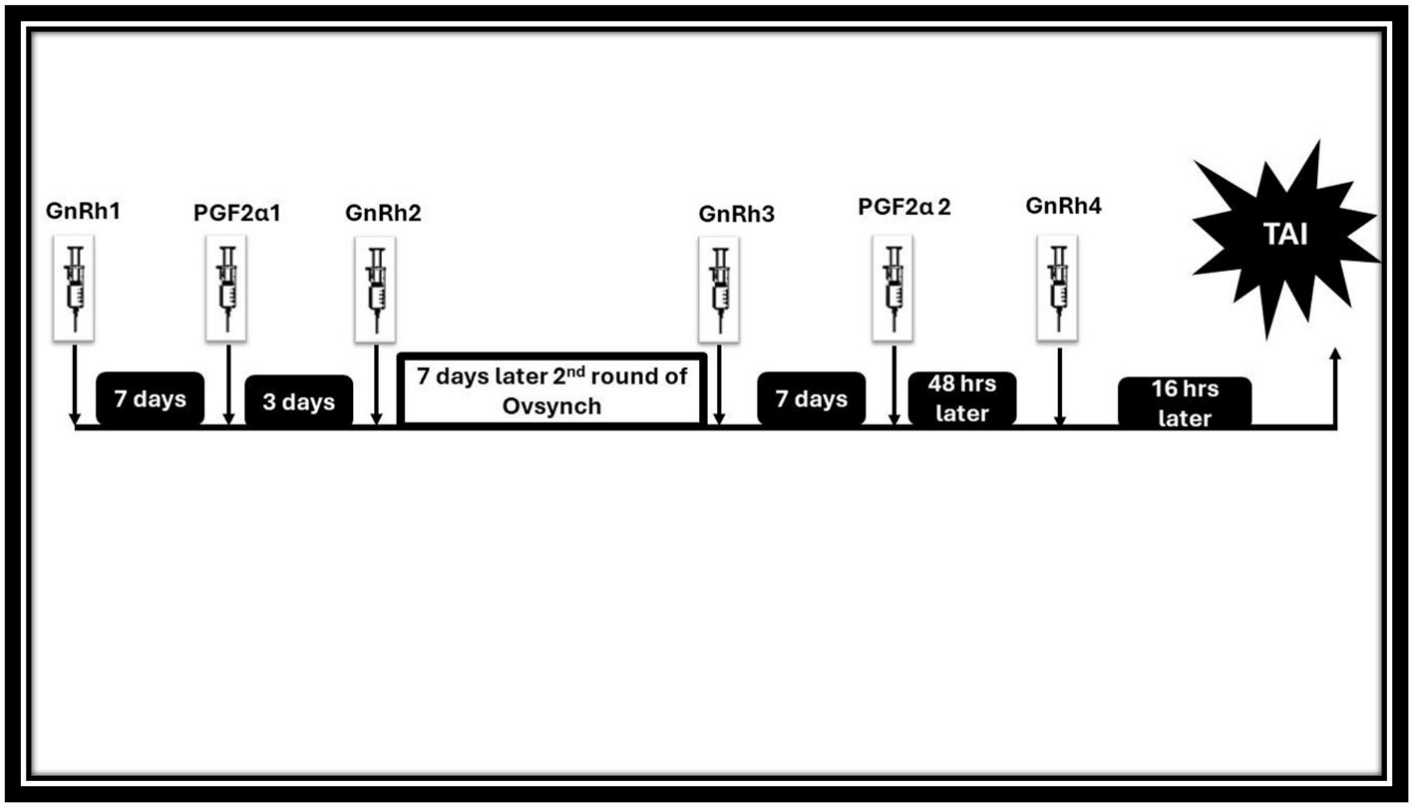
GnRH based treatment (Double Ovsynch).

Double-Ovsynch protocols involve two rounds of the Ovsynch protocol to improve synchronization and conception rates (89, 218). The FTAI is performed at a precisely scheduled time following the second GnRH of the second Ovsynch sequence as illustrated in Figure 7 (204, 219). Incorporating GnRH into a pre-synchronization strategy, as implemented in protocols like Ovsynch1, enhances conception rates by targeting the anovular state often observed in cows prior to starting the main Ovsynch protocol (220). The Double Ovsynch protocol resulted in a notably higher conception rate among primiparous cows, achieving 44%, compared to 31% in multiparous cows. This demonstrates the effectiveness of the protocol, particularly in younger, first-time calving cows (221). This protocol has proven effective as a resynchronization protocol, achieving higher conception rates compared to the standard Ovsynch (39% vs. 30%) in cattle. In acyclic heifers and buffaloes a higher conception rate of 83.33% was investigated after using double Ovsynch protocol in Indian Gujrat (222). This effectiveness hinges on having sufficient time to complete the entire protocol without excessively increasing the number of days open (223).

### 4.2 Factors affecting FTAI in buffaloes

Timed Artificial Insemination in buffaloes relies on a complex interplay of factors to determine FTAI success. This reproductive technique involves synchronization of estrus, precise insemination, and favorable environmental and health conditions for the buffalo. The efficacy of FTAI is influenced by several key factors that encompass hormonal synchronization protocols, the skill of the inseminator, the reproductive health of the buffalo, and environmental elements such as nutrition and stress levels. A thorough understanding and management of these factors are critical in optimizing the success rates of FTAI in buffalo breeding programs. There are several key factors that can influence its success in buffaloes.

#### 4.2.1 Estrous synchronization protocols

Proper timing and administration of hormonal protocols/interventions to synchronize estrus and ovulation increase the likelihood of inseminating buffaloes during their fertile period (224). Estrous synchronization is pivotal in timed artificial insemination, aligning the reproductive cycle of buffaloes for optimal conception (225, 226). Hormonal protocols regulate estrus, ensuring the female buffaloes are at a prime stage for successful insemination. Proper timing and administration of hormones are crucial for synchronization, enhancing the chances of successful breeding (39). How the estrous synchronization protocol is a major influencing factor in FTAI and has yielded varying conception rates is summarized in Table 4.

**Table 4:** Comparison of conception rates across different synchronization protocols in female buffaloes.

**Protocol**	**Season**	**Breed**	**Conception rate %**	**References**
1. Double synch: Day treatment start i.e., 0: PGF2α injected, Day-2: GnRH, Day 9: PGF2alpha, Day-11: GnRH (TAI after 16 and 24 h after GnRH)	Breeding	Murrah	60	(269)
2. Estradiol + CIDR: Day 0: E_2_+CIDR for 4 days, Day 9: CIDR was removed and PGF2alpha was injected. Day 11: GnRH (AI after 12 h), Day-12: 2nd time AI	Breeding	Murrah	48	(270)
3. Modified Co-synch: Day treatment start 0: GnRH, Day 3: PMSG, Day7: PGF2 α, Day-9: hCG/TAI	Non-breeding	Murrah	53.8	(271)
4. Modified Co-synch: Day protocol start- 0: GnRH+ CIDR, Day-3: PMSG, Day-7: PGF2alpha, Day-9: hCG/TAI	Non-breeding	Murrah	33.8	(271)
5. GPGMH (modified Ovsynch): Day-0: GnRH, Day 7: PGF2alpha, Day-9: 2nd time GnRH+ mifepristone, Day-10: AI and on Day 15: hCG	Breeding	Crossbreed	47.1	(7)
6. Estradiol benzoate+ CIDR: Day 0: Estradiol benzoate, Day 9: P_4_ removal and PGF2α+ eCG administered IM. Day 11: GnRH and Day 12: TAI	Breeding	Crossbreed	60–68	(92)
7. Insulin modified ovsynch: Day start 0: GnRH, Day- 7: PGF2α, Day- 9: GnRH (same time long-acting biphasic Insulin subcut), Day 10: Insulin, Day 11: Insulin (TAI 12–14 h after GnRH), Day-11: TAI	Breeding	Murrah	73.33	(180)
8. CIDR Cosynch: Day 0–7: CIDR, Day 7: CIDR removed and PGF2α injected, Day 10: GnRH (After 84 h TAI)	Breeding	Nili-Ravi	65	(201)
9. G6G Ovsynch: Blind PGF2α injection, followed by GnRH after a 2-day interval. 06 days later started the OV-synch protocol	Breeding	Egyptian	61	(89)
10. Modified fixed time AI (Ovsynch): Day start protocol 0: GnRH, Day-7: PGF2 α, Day-9: Ultrasound + GnRH, Day-10: US+ unicornual FTAI only in buffaloes with dominant follicle	Breeding	Romanian	63.6	(183)

#### 4.2.2 Reproductive management

Good reproductive management practices, including proper nutrition, health management, and regular monitoring of estrus behavior, are essential for FTAI success (227, 228). Body condition score (BCS) has an association with productive pregnancy especially during the poor breeding season (229). Farmers need to be aware about such buffaloes exhibiting a low BCS may not exhibit positive responses to various protocols (230). Some authors believe that body condition influence the ovarian cycle of bovine family directly and found an upsurge in the reproductive ovarian cycle of bovine, corresponding to an improvement in the body condition (41, 231). It is advisable to promote efforts aimed at enhancing the nutritional status of the animals ensuring optimal body condition and addressing any reproductive disorders or health issues that can improve the chances of successful AI (173, 232).

#### 4.2.3 Semen quality, AI technique, and genetic factors

The quality of the semen used for FTAI significantly impacts the conception rates (233, 234). Using high-quality semen from genetically superior bulls with good fertility is crucial for maximizing the chances of successful AI in buffaloes (34, 235). An increase in the conception rate up to (63%) using FTAI protocol in Romanian buffaloes was observed by using sexed semen containing 2 million X-chromosome bearing sperm (183). The proficiency and skill of the inseminator in performing AI techniques are crucial for FTAI success (236). Different buffalo breeds may exhibit variations in reproductive characteristics and responses to FTAI protocols. Additionally, genetic factors, including the genetic potential for fertility and reproductive traits, can influence the success of FTAI (229).

#### 4.2.4 Season and stress levels

Buffaloes become sexually activated in response to decreased day length and temperature (237). Seasonality in water buffalo extends postpartum anestrus intervals, adversely impacting the reproductive performance (33). The efficiency of ovulation synchronization protocols for FTAI can be affected by the reproductive seasonality observed in buffaloes (91). During the seasonal anestrus period, buffaloes experience a lack of behavioral estrous, ovulation, and decreased progesterone secretion (238). Consequently, there is an occurrence of ovarian follicular turnover during this time (239). Hormonal treatment can be employed to persuade estrus or ovulation in anestrous cows (41). However, certain buffaloes never respond to the treatment due to low breeding season (111). Various factors may contribute to this phenomenon, but one of the most probable reasons is the follicular status of the animal at the initiation of FTAI protocol (51). The ideal time for treatment can be determined via ovarian activity with ultrasound (240). Reproductive efficacy depends on the breeding season and high conception rate (129, 241). Buffaloes exhibit a seasonal breeding pattern and typically develop sexual activity in response to a decreasing day length, which occurs during later summer to early autumn (9, 242). The breeding season influences the conception rate (50). Buffalo cows treated throughout the breeding season (autumn and winter) shown a higher conception rate compared to those treated throughout the off-breeding season, with rates of 48.8% (472/967) and 6.9% (6/86) respectively (41, 147).

High stress levels, caused by factors such as transportation, handling, or changes in the environment (229), can negatively impact reproductive performance in buffaloes. Heat stress adversely influences the reproductive performance as well as production of Buffalo (243). Buffalo is more vulnerable to heat stress than cattle due to fewer sweat glands and black hair resulting in fertility loss (244). When buffaloes experience heat stress, their consumption and efficiency in utilizing feed are reduced, leading to alterations in the balance of proteins, water, energy, and minerals. Additionally, changes in enzymatic activities and hormone levels negatively impact buffalo reproduction (245). The temperate zone is regarded as the most conducive for enhanced productivity in dairy animals (246). Minimizing stressors and providing a calm and conducive environment can improve FTAI success (247).

#### 4.2.5 Timing in FTAI

Timing is critical in FTAI, as it determines the optimal moment for insemination (26). Insemination is typically performed 48–72 h after the withdrawal of progesterone supplementation or prostaglandin administration when ovulation is expected to occur (52, 248). This allows for the delivery of sperm to the reproductive tract during the fertile window, maximizing the chances of successful fertilization (249). The implementation of FTAI in buffalo breeding programs offers several advantages. It eliminates the need for estrus detection, which can be challenging in buffaloes due to the absence of clear behavioral signs (250). FTAI also allows for the efficient utilization of superior sires by precisely timing insemination (39) and improving the genetic potential of the offspring (251). Additionally, it can enhance reproductive management by enabling breeders to optimize breeding programs, synchronize calving intervals, and increase overall herd productivity (252). These factors can affect the response to hormonal treatments, the synchronization of estrus, and the overall fertility rates. Understanding and managing these factors are crucial for improving timed artificial insemination outcomes in buffalo breeding programs (253).

## 5 Concluding remarks and suggestions

Fixed-time artificial insemination (FTAI) holds immense potential for optimizing buffalo reproduction, yet its efficacy remains uneven across breeds and seasons. We have discussed various possibilities to initiate and maintain FTAI program under different circumstances according to the specific reproductive physiology of the buffalo and the factors affecting TAI efficiency. As summarized in our review paper's Table 4, slight modifications in ovsynch protocol with FTAI resulted in high conception rate during breeding season but in the protocol no. 5 conception rate was not so high possibly due to cross breed and climate factor or due to genetic differences in follicular dynamics. Insulin modified protocol could be a better choice for crossbreed buffaloes. For cost effective TAI protocol CIDR can be replaced with biodegradable progesterone implants. However, to reduce human error and to refine ovulation window integrated ultrasound-guided FTAI and 5 days later FTAI single hCG shot will be a best possible choice for FTAI. Notably, still there is not any single magical tool or protocol. Therefore, certain adjustments and possible factors affecting FTAI efficiency like season, nutritional, health status, breed of animal and cost of the protocol should be considered. Finally, better understanding of buffalo's reproductive physiology and considering the elements that impact on the effectiveness of FTAI can reinforce the likelihood to achieve better outcomes or provide clearer insight when the results differ from our expectations.
